# Collaboration of perioperative management in an adult patient with 22 q 11.2 deletion syndrome: A case report

**DOI:** 10.1002/ccr3.5489

**Published:** 2022-02-18

**Authors:** Mikiko Ito, Tatsuya Tokura, Tomoya Miyauchi, Aiji Sato (Boku), Hiroyuki Kimura, Hayami Tsuchihashi, Yoshiko Katayama

**Affiliations:** ^1^ Department of Oral and Maxillofacial Surgery, School of Dentistry Aichi‐Gakuin University Nagoya Japan; ^2^ Department of Psychiatry Nagoya University Graduate School of Medicine Nagoya Japan; ^3^ Department of Psychiatry Kachi Memorial Hospital Toyohashi Japan; ^4^ Department of Anesthesiology, School of Dentistry Aichi‐Gakuin University Nagoya Japan

**Keywords:** 22 q 11.2 deletion syndrome, multidisciplinary team approach, perioperative management

## Abstract

A 24‐year‐old woman diagnosed with 22 q 11.2 deletion syndrome was referred for multiple extractions. Due to the syndrome, the patient had schizophrenia, cardiac anomalies, and maxillofacial complications. This case report suggested that a multidisciplinary team approach is important for perioperative management of patients with 22 q 11.2 deletion syndrome.

## Graphical abstract

A 24‐year‐old woman diagnosed with 22 q 11.2 deletion syndrome was referred for multiple extraction. The patient had schizophrenia, cardiac anomalies, and maxillofacial complications which required a multidisciplinary team approach.

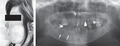

## 1 INTRODUCTION

22 q 11.2 deletion syndrome (22 q 11.2 DS) is a genetic disease that is often complicated by psychiatric disorders such as intellectual disability, autism spectrum disorder, attention‐deficit/hyperactivity disorder, and schizophrenia in addition to physical diseases such as congenital heart disease, palate hypoplasia, and immunodeficiency. This syndrome is said to be the most common of the chromosomal microdeletion syndromes, and a recent Danish cohort study reported that 1 in 3672 people developed it.^1^ Although the prognosis has been said to be poor, the adult population with 22 q 11.2 DS is increasing due to advances in pediatric care.^2^

22 q 11.2 DS was first described as DiGeorge syndrome in the early 1960s. Initially, DiGeorge syndrome was noted for its frequent complications of oral diseases.^3^, ^4^ Subsequently, disease units such as velo‐cardio‐facial syndrome and conotruncal anomaly face syndrome were proposed, but these were a group with various clinical symptoms and different severity. Recently, similar deletions of similar genomic regions have been found in patients with these disease units, and the clinical series resulting from the 22 q 11.2 deletion is now referred to as 22 q 11.2 DS.^5^, ^6^

Since multiple diseases often coexist in 22 q 11.2 DS, multiple departments are involved in treatment and medical cooperation is extremely important. Acceptable standard care for individuals with 22 q 11.2 DS is a careful coordination and multidisciplinary team approach that provides access to medical services throughout their life cycle.^7^ In this report, we successfully collaborated in the perioperative management of oral surgery in an adult 22 q 11.2 DS patient with multiple physical diseases and schizophrenia.

## 2 CASE REPORT

The patient was a 24‐year‐old woman. The patient was aware of pain from cold water and pain in the whole jaw during chewing. However, the patient was unable to successfully complain of pain due to mental disorder. The patient visited our oral surgery department accompanied by mother with the chief complaint of masticatory disorder.

**FIGURE 1:**
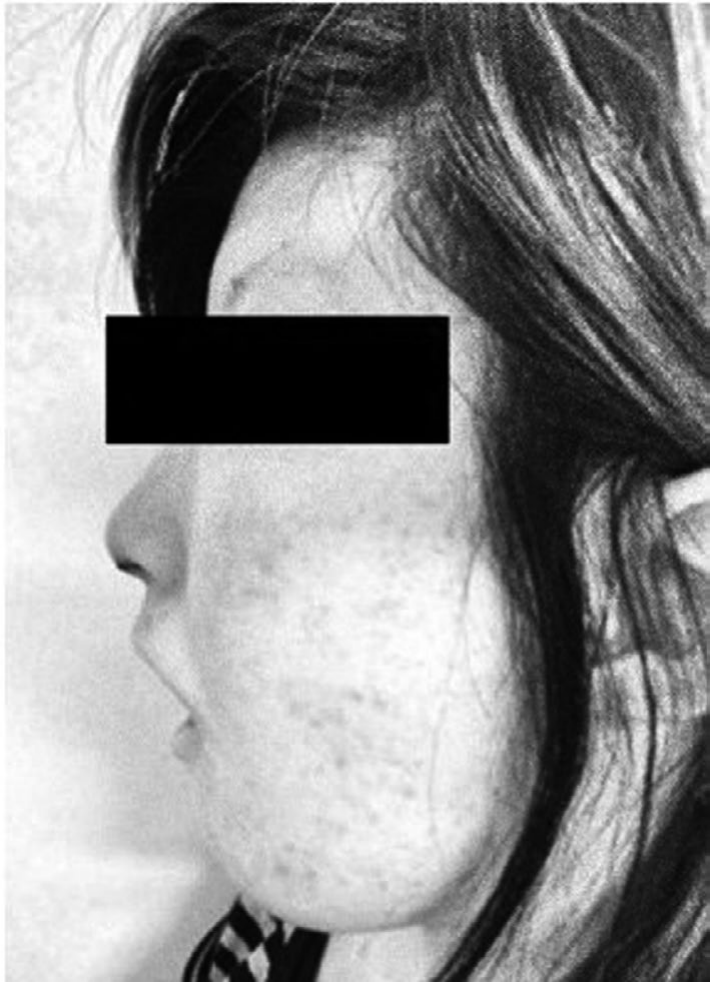
Facial appearance findings. Microstomia, micronostrils, and micrognathia were observed

**FIGURE 2:**
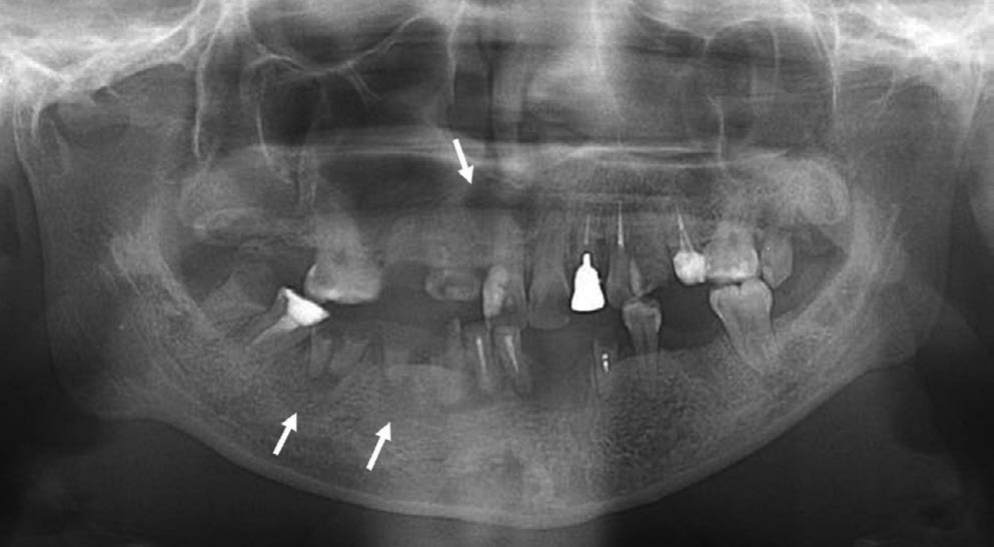
Panoramic X‐ray photograph at the first visit. Panoramic radiographs showed the apices of 11, 45, and 46 (Fédération Dentaire Internationale) with round radiolucent images suggesting apical lesions (Arrows to the lesions)

Extraoral findings of the patient showed microtia, micromandibula, micronostrils, micrognathia, and trismus (Figure 1). The intraoral findings showed that 15, 17, 28, 34, 42, 43, 45, 46, and 48 (Fédération Dentaire Internationale, FDI) were in a residual root state. The patient's oral hygiene status was extremely poor. Panoramic radiographs showed the apices of 11, 45, and 46 (FDI) with round radiolucent images suggesting apical lesions (Figure 2).

The patient was 148 cm tall, weighed 41 kg, and had a blood pressure of 90/60 mmHg, heart rate 110/minutes at the time of first medical examination. There were no other problems with blood tests.

The patient was born at 39 weeks and 2 days of gestation. Anal atresia and cleft palate were found at birth. At one month after birth, the patient was diagnosed as having aortic stenosis due to bicuspid aortic valve in the pediatric cardiology department, and diagnosed with 22 q 11.2 DS by genetic testing. The patient underwent palatoplasty at the age of 1 year and 6 months, and orthodontic treatment from the age of 9 to 20 years with a diagnosis of dental stenosis. The patient had been followed up at the pediatric cardiology department for bicuspid aortic valve and aortic stenosis, which had been discovered at birth. The patient attended regular classes at elementary and junior high schools. However, the patient was bullied by her classmates, and self‐injurious behavior such as cutting the wrist and picking the nails was recognized. After graduating from high school, the patient worked as a care worker for three and a half years. At the age of 21, the patient got married and gave birth by performing a cesarean section. At the age of 22, the patient was diagnosed with schizophrenia due to the patient's hallucination and delusion and was admitted to a psychiatric hospital for 10 months. The symptoms have improved by taking antipsychotic drug and modified electroconvulsive therapy.

We planned to extract 10 teeth, specifically (11, 15, 17, 28, 34, 42, 43, 45, 46, and 48 [FDI]) under general anesthesia. The risk of multiple extraction itself was low, but the risks arising from complications of 22 q 11.2 DS were a concern. Hypotension and sinus tachycardia were observed during an examination by an internist in our hospital. Aortic regurgitation was mild in the echocardiogram, and it was judged as an indication for general anesthesia. Although there were no problems with cardiac function, the patient was at risk for infective endocarditis, and 2 g of ampicillin was prepared.

The dental anesthesiologist diagnosed the patient as having difficult intubation due to micrognathia and severe trismus and judged that conscious fiberscope‐guided intubation under mild sedation was also impossible because the patient's psychiatric symptoms were unstable. After induction of general anesthesia, the dental anesthesiologist administered 30 mg of rocuronium, a muscle relaxant, and performed nasotracheal intubation under fiber scope guidance. We explained the risks of anesthesia to the patient and the patient's family in advance and obtained their consent in writing.

The following risks information were provided by the attending psychiatrist: 1) discontinuation of psychotropic drugs may aggravate schizophrenia; 2) examination by an oral surgeon may increase anxiety and irritation; and 3) interactions between antipsychotics and epinephrine contained in the local anesthetics may occur. Therefore, the administration of psychotropic drugs was continued even on the night before the operation, the preoperative examination by the oral surgeon was shortened, and the local anesthetic for surgery was prepared as ferripressin‐containing propitocaine hydrochloride without epinephrine.

The above risks were shared by a multidisciplinary team including oral surgeons, dental anesthesiologists, psychiatrists, cardiologists, nurses, and social workers. We extracted 10 teeth as scheduled. The day after the operation, we transferred the patient to the psychiatric hospital where the patient had been hospitalized. One week later, the patient had good progress, and we completed the collaboration of perioperative management.

## 3 DISCUSSION

The course of this patient provides the following important clinical suggestions. Since multiple physical diseases and psychiatric disorders often coexist in 22 q 11.2 DS, a multidisciplinary team approach is important for perioperative management of patients with 22 q 11.2 DS.

Frequent comorbidities of 22 q 11.2 DS are multidisciplinary, including cardiac anomalies (49%–83%), palatal anomalies (69%–100%), dental skeletal anomalies (40%–50%), hypocalcemia (17%–60%), schizophrenia (6%–30%), and attention‐deficit/hyperactivity disorder (25%).^5^

Life‐threatening complications include congenital heart disease and the most common one is tetralogy of Fallot (20%–45%).^6^ Congenital heart disease adds to the complexity associated with acquired cardiovascular problems and multiorgan comorbidities, and requires perioperative management that considers immune dysfunction, thrombocytopenia, and hypocalcemia.^8^ This patient had no tetralogy of Fallot but aortic stenosis. Aortic stenosis is a rare complication of 22 q 11.2 DS,^5^ but it is a risk group for infective endocarditis. Therefore, antibiotic ampicillin was administered prophylactically.

In general, physicians have trouble dealing with patients with psychiatric disorders, while psychiatrists have trouble dealing with patients with physical illness. Perioperative management of oral surgery for patients with schizophrenia requires a history of psychiatric treatment, assessment of psychotic symptoms, and consideration of the duration of psychotropic drug withdrawal to control schizophrenic symptoms.^6^, ^9^ This case was also coordinated to be transferred to the psychiatric hospital where the psychiatrist worked the day after the operation in accordance with the risk information obtained from the psychiatrist.

In 22 q 11.2 DS, a high rate of palatal hypoplasia is observed, and it is necessary to devise measures for difficult intubation. We decided that it would be difficult to intubate this patient due to micrognathia and trismus. The patient had experienced general anesthesia in the past. At that time, oral intubation by laryngeal mask was performed, because the operative field was not the oral cavity. We finally chose nasofiberscope‐guided intubation after induction of general anesthesia. In patients with 22 q 11.2 DS, general anesthesia or intravenous sedation has been used for dental treatment.^10^ However, there have been no previous reports of fiberscope‐guided nasal intubation after induction of general anesthesia. Our team responded flexibly to risks with our own ideas, using precedents as a guide.

We reported an adult patient with 22 q 11.2 DS in whom a multidisciplinary team approach was effective in collaboration with perioperative management. In the future, it is expected that the number of adult patients with 22 q 11.2 DS who require oral surgery under general anesthesia with multiple risks will increase as in this case. Since the symptoms and severity of 22 q 11.2 DS vary widely among individuals, it is suggested that accumulation of individual perioperative management reports may be clinically useful in the future.

## 4 CONCLUSION

We reported an adult patient with 22 q 11.2 DS who needed to extract teeth. Through our report with this case, we concluded that a multidisciplinary team approach is important for perioperative management of adult patient with 22 q 11.2 DS.

## CONFLICT OF INTEREST

There were no conflict of interest related to this publication.

## AUTHOR CONTRIBUTIONS

All authors contributed to the case. Preoperative management was performed by YK, MI, and HT. Anesthetic planning and management were performed by AS. The first draft of the manuscript was written by MI, TM, and HK, and all authors commented on previous versions of the manuscript. TT supervised the manuscript. All authors read and approved the final manuscript.

## CONSENT

Written informed consent was obtained from the patient and the patient's family for this case report.

## Data Availability

Not applicable.
